# Risk of re-attempts and suicide death after a suicide attempt: A survival analysis

**DOI:** 10.1186/s12888-017-1317-z

**Published:** 2017-05-04

**Authors:** Isabel Parra-Uribe, Hilario Blasco-Fontecilla, Gemma Garcia-Parés, Luis Martínez-Naval, Oliver Valero-Coppin, Annabel Cebrià-Meca, Maria A. Oquendo, Diego Palao-Vidal

**Affiliations:** 1 Department of Mental Health, Parc Tauli-University Hospital, Parc Taulí 1, 08208 Sabadell, Barcelone Spain; 2 Department of Psychiatry and Forensic Medicine, Universitat Autònoma de Barcelona, Barcelone, Spain; 3 Department of Psychiatry, IDIPHIM-Puerta de Hierro University Hospital, Autonoma University of Madrid, Avenida Manuel de Falla s/n, Madrid, Spain; 4 CIBERSAM, Madrid, Spain; 5 Department of Mental Health, Meritxell Hospital, Andorra la Vella, Andorra; 6 Institute of Legal Medicine of Catalonia, Barcelone, Spain; 7 Statistical Department, Universitat Autònoma de Barcelona, Barcelona, Spain; 8 Perelman School of Medicine, University of Pennsylvania, Pennsylvania, USA

**Keywords:** Suicidal behaviour, Alcohol use disorders, Personality disorders

## Abstract

**Background:**

Suicide is the primary cause of unnatural death in Spain, and suicide re-attempts a major economic burden worldwide. The risk factors for re-attempt and suicide after an index suicide attempt are different.

This study aims to investigate risk factors for re-attempt and suicide after an index suicide attempt.

**Methods:**

This observational study is part of a one-year telephone management program. We included all first-time suicide attempters evaluated in the emergency department at *Parc Taulí-University Hospital* (*n* = 1241) recruited over a five-year period (January 2008 to December 2012). Suicide attempters were evaluated at baseline using standardized instruments. Bivariate logistic regression models were used to identify risk factors. Kaplan-Meier curves were used to compare the time to re-attempt between categorical variables. Comparisons were performed using Log-Rank and Wilcoxon tests. Variables with a *p*-value lower than 0.2 were included in a multivariate Cox regression model. Bivariate logistic regression models were considered to identify risk factors for suicide. The significance level was set to 0.05.

**Results:**

Suicide re-attempters were more likely diagnosed with cluster B personality disorders (36.8% vs. 16.6%; *p* < 0.001), and alcohol use disorders (19.8 vs. 13.9; *p* = 0.02). Several [1.2% (15/1241)] of them died by suicide. Attempters who suicide were more likely alcohol users (33.3% vs. 17.2%; *p* = 0.047), and older (50.9 ± 11.9 vs. 40.7 ± 16.0; *p* = 0.004).

**Conclusions:**

Alcohol use, personality disorders and younger age are risk factors for re-attempting. Older age is a risk factor for suicide among suicide attempters. Current prevention programs of suicidal behaviour should be tailored to the specific profile of each group.

## Background

Suicide is a global health issue and since 2008, it is the primary cause of unnatural death in Spain [1]. A history of previous suicide attempt is the strongest predictor for future suicidal ideation and behaviour (SIB), including suicide ideation, suicide attempts, and suicide [2–5]. For instance, in a 5 years follow-up of 302 individuals admitted to an inpatient psychiatric unit for medically serious suicide attempts, 37% of them made at least one further suicide attempt, and 6.7% eventually died by suicide [6]. Furthermore, most suicides occur in people with mental disorders [1], but most people with mental disorders, even severe, never attempt suicide [7]. In other words, this risk factor and many others have poor predictive power. Therefore, a better differentiation between suicide attempters who eventually suicide and suicide attempters who will not is critical to developing preventive plans.

In a systematic review of 14 cohorts (*n* = 21,385), Neeleman estimated that individuals with antecedents of self-harm were 25 times more likely to die by suicide than the general population [8]. Owens et al. [9] reviewed 80 observational and empirical studies and concluded that the risk of another SIB ranged between 16% (first year) and 23% (follow-up of 4 years or longer), whereas for suicide it ranged from 2% (first year) to 7% (follow-up of 9 years). Christiansen et al. [10] estimated the risk of another SIB in a five-year follow-up study at about 31%. These authors stressed that the risk of another SIB was higher during the first two-years after the index suicide attempt. Female gender and the presence of mental disorders are well-known risk factors for repeated SIB [10]. Other authors have stressed the role of personality disorders, particularly borderline personality disorder, in future SIB [11]. On the other hand, between 1 and 6% of individuals evaluated because of a suicide attempt eventually suicide in the year following. The risk of suicide is higher in older patients and those individuals with a higher number of lifetime suicide attempts [12–15], counter to clinical lore about frequent attempters not being at risk for suicide because they only engage in low risk SIB.

Even if evidence is scarce, recent studies have demonstrated that it is possible to reduce the risk of re-attempt or even suicide in individuals at risk [16, 17]. For instance, we previously reported that a one-year telephone intervention program was effective in reducing an 8% the proportion of patients who re-attempted suicide compared to the control population [18]. This is in keeping with some [19] but not all [20] previous literature on the effectiveness of telephone intervention programs.

Aims of the study: The main objective of the current study is to identify risk factors for re-attempt and suicide using survival analysis.

## Method

### Samples and procedure

This observational study is part of a one-year telephone management program, which forms part of the European Alliance Against Depression (EAAD) framework for the management of SIB [17]. All first-time suicide attempters (index suicide attempt) evaluated in the emergency department (ED) at *Parc Taulí-University Hospital*, Spain (*n* = 1241) between January 1st 2008 and December 31st 2012 were approached to take part in a one-year telephone follow-up prevention program that had the objective of reducing suicide attempts rate [18]. This telephone management program was aimed at determining the effectiveness over 1 year of a follow-up on patients discharged from the ED after a suicide attempt. The one-year telephone intervention program reduced an 8% the proportion of patients who re-attempted suicide [18].

This ED sees all medical emergencies for a catchment population of 474,778 inhabitants. On-call psychiatrists evaluated all suicide attempters. A suicide attempt was defined as a self-harming behaviour with clear suicidal intent [21]. All suicides (*n* = 142) in our hospital’s catchment area were recorded during this period of time, based on direct information from the Institute of Forensic Medicine of Catalonia, charged with making determinations about cause of death.

The primary outcome measures were time to new suicide behaviour (SB; either suicide attempt or suicide, only suicidal ideation was not included), and the percentage of suicide attempters who re-attempted suicide or suicide during the period of study. The information on re-attempts was extracted from the electronic medical record. The Institute of Forensic Medicine of Catalonia provided information on suicide deaths. All first-time suicide attempters recruited during the last year (1st January 2012 to December 31st 2012) were equally offered the 1-year telephone follow-up (up to December 31st 2013). Accordingly, the information on the main outcomes of our study (re-attempts and suicides) ranges from 1 to 6 years.

All first time suicide attempters provided information on sociodemographic factors (sex, age, marital status, place of birth, level of education, employment status, and living arrangements), clinical factors (multiaxial psychiatric diagnosis according to DSM-IV-TR criteria, previous medical follow-up), characteristics related to the suicide event (method used, date of the attempt, consumption of drugs or alcohol at the time of the act, and degree of lethality (mild: < 24 h in the ED for medical observation/intervention; moderate: 24–48 h in the ED; severe: > 48 h in the ED or surgical intervention or psychiatric inpatient hospitalization), and type of medical follow-up prior to the SB. Data were obtained from inpatient clinical histories and from emergency and primary care electronic reports.

All first time suicide attempters discharged from the ED were scheduled for a post-discharge visit with the referring psychiatrist within a maximum of 10 days and verbally consented to participate in a telephone follow-up during a year. The telephone follow-up was conducted by a nurse specialized in mental health who had received specific training on the administration of the program, detection of high risk for suicide and management of patients with low and mild risk of suicide. The telephone follow-up was carried out at 1 week, 1 month and, thereafter at 3, 6, 9 and 12 months after the index suicide attempt. Further information can be found elsewhere [18].

We confirmed that all individuals were seen either in our mental health center or in the primary care center for at least 1 year after the end of study enrolment (December 31st 2012). Whenever this information was not available, a phone call confirmed that the individual was alive and did not change their place of residence.

**Fig. 1:**
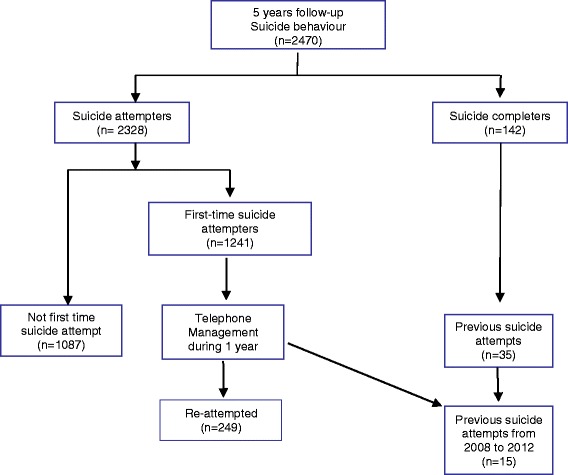
Progress of participants through trial

The progress of all participants through the study is detailed in Fig. 1.

### Statistical analysis

Descriptive statistics of socio-demographic characteristics at the index suicide attempt are presented for re-attempters, non re-attempters and globally (absolute and relative frequencies). Bivariate logistic regression models were used to identify risk factors [22] and odds ratios (OR) and 95% confidence intervals were calculated.

Kaplan-Meier curves for all variables -sociodemographic and clinical factors, and characteristics related to the suicide event- were used to compare the time to re-attempt between re-attempters and non re-attempters. Comparisons were performed using Log-Rank and Wilcoxon tests. Variables with a *p*-value lower than 0.2 were included in a multivariate Cox regression model [23]. Hazard ratios (HR) and 95% confidence intervals were calculated.

Additionally, bivariate logistic regression models were constructed to identify risk factors for suicide. Given the small number of suicides, we could not run multivariate analyses in the case of suicides.

The analysis was performed with software SAS v9.3 (SAS Institute Inc., Cary, NC, USA). Alpha was set to 0.05.

## Results

### Sociodemographics

Suicide attempts represented 0.3% of all emergencies presenting to the ED during the study period and 14.4% of psychiatric emergencies. The suicide rate in our catchment area was 8.3/100,000 inhabitants in 2008, 6.6/100,000 in 2009, 7.2/100,000 in 2010, 4.8/100,000 in 2011 and 7.2/100,000 in 2012. During the 5 years of recruitment, there were 2328 suicide attempts made by 1627 patients evaluated at *the Parc Taulí Sabadell-University Hospital*. From that sample, we selected first-time suicide attempters (*n* = 1241). Women represented 62.4% of our sample, and the mean age was 40.8 (±16.0). The most frequent method used in the index suicide attempt was drug overdose (70.8%). Around 20% (20.5%) were hospitalized in the acute mental health unit. Table 1 displays socio-demographic characteristics at the index suicide attempt.

**Table 1:** Distribution of socio-demographic characteristics at the index suicide attempt

	No re-attempters (*n* = 992)		Re-attempters (*n* = 249)		Total (*n* = 1241)		*p*-value	O.R. (95% CI)
	n	%	n	%	n	%		
Sex	992	100	249	100	1241	100	*p* = 0.213	
Male	381	38.4	85	34.1	466	37.6		**-**
Female	611	61.6	164	65.9	775	62.4		1.16 (0.92–1.47)
Age	992	100	249	100	1241	100	*p* = 0.025	
< 20	71	7.1	21	8.4	92	7.4		2.03 (1.14–3.61)
20–29	184	18.5	43	17.3	227	18.3		1.68 (1.01–2.81)
30–39	246	24.8	75	30.1	321	25.9		2.08 (1.29–3.35)
40–49	211	21.3	63	25.3	274	22.1		2.04 (1.26–3.32)
50–59	138	13.9	29	11.6	167	13.4		1.54 (0.89–2.67)
≥ 60	142	14.3	18	7.2	160	12.9		-
Marital status	772	100	197	100	969	100	*p* = 0.161	
Unmarried	157	20.3	48	24.4	205	21.1		3.04 (0.99–9.30)
Stable partnership/married/cohabiting	404	52.3	100	50.8	504	52		2.58 (0.86–7.77)
Separated/Divorced	175	22.7	46	23.3	221	22.8		2.71 (0.88–8.28)
Widower	36	4.7	3	1.5	39	4		-
Place of birth	949	100	240	100	1189	100	*p* = 0.226	
Spain	834	87.9	219	91.2	1053	88.6		1.75 (0.86–3.55)
South America	63	6.6	14	5.8	77	6.5		1.53 (0.66–3.56)
Other	52	5.5	7	2.9	59	4.9		-
Educational level	278	100	72	100	350	100	*p* = 0.677	
Primary	142	51.1	34	47.2	176	50.3		1.35 (0.52–3.53)
Secondary I	77	27.7	23	31.9	100	28.6		1.61 (0.60–4.28)
Secondary II	35	12.6	11	15.3	46	13.1		1.67 (0.59–4.77)
College	24	8.6	4	5.6	28	8		-
Employment situation	625	100	160	100	785	100	*p* = 0.306	
Employed	208	33.3	57	35.6	265	33.8		1.45 (0.95–2.20)
Unemployed	132	21.1	36	22.5	168	21.4		1.44 (0.92–2.27)
Pensioner	155	24.8	27	16.9	182	23.2		-
Student	51	8.2	16	10	67	8.5		1.61 (0.93–2.80)
No income	79	12.6	24	15	103	13.1		1.57 (0.96–2.58)
Home living arrangements	633	100	168	100	801	100	*p* = 0.621	
Living alone	87	13.7	21	12.5	108	13.5		-
1 person	170	26.8	38	22.6	208	25.9		0.94 (0.58–1.52)
2 people	201	31.7	63	37.5	264	32.8		1.23 (0.79–1.91)
3 people	120	19	32	19	152	18.9		1.08 (0.66–1.77)
≥ 4 people	55	8.7	17	10.1	72	9.0		1.21 (0.69–2.14)

Two hundred and forty-nine (20.1%) of first-time suicide attempters, re-attempted suicide at least once, and 15 (1.2%) died by suicide during follow-up (mean and median time of follow-up for re-attempts and suicides were 298 and 177 days, respectively). Here, it is important to stress that during this period, of the 142 suicides in our catchment area, 127 [87.5%; *n* = 89 (70.1%) males, and *n* = 38 (29.9%) women] were not evaluated in the ED, even though 35 [24.6%; 18 women (12.6%) and 17 men (12%)] of them had a previous suicide attempt. Of those 35 patients, 15 patients had previous suicide attempts evaluated in the ED during the follow-up, and the remaining 20 patients had attempted suicide before the follow-up (see Figure 1).

### Timing of the survival curve for re-attempts and suicide

**Fig. 2:**
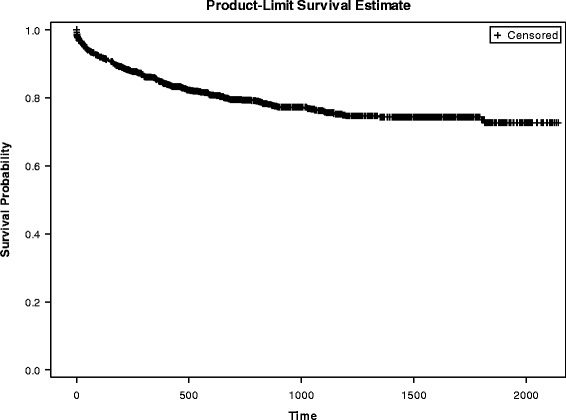
Survival estimates (re-attempts)

**Fig. 3:**
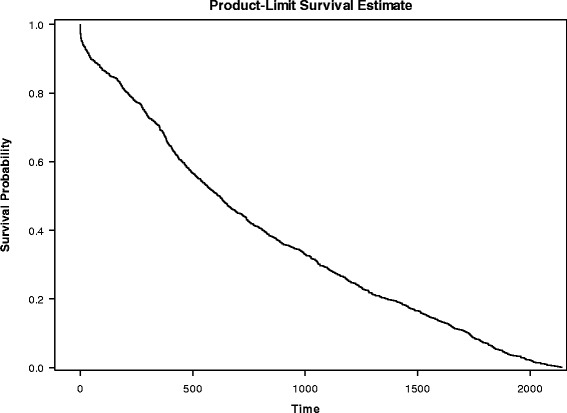
Survival estimates (completed suicide)

Most (88%) re-attempts and suicides (93%) took place within the first-2 years of follow-up (see Tables 2 and 3, and Figs. 2 and 3). Figures 2 and 3 display the survival curve of re-attempts and completed suicides, respectively.

**Table 2:** Proportion of first-time suicide attempters who re-attempted suicide at 2 years follow-up

Follow-up >2 years	Re-attempt		
N % (col)	Yes	No	Total
Yes	29	517	546
	11.65	52.12	
No	220	475	695
	88.35	47.88	
Total	249	992	1241

**Table 3:** Proportion of first-time suicide attempters who suicide at 2 years follow-up

Follow-up >2 years	Suicide		
N % (col)	Yes	No	Total
Yes	1	545	546
	6.67	44.45	
No	14	681	695
	93.33	55.55	
Total	15	1226	1241

### Risk factors for re-attempts

**Fig. 4:**
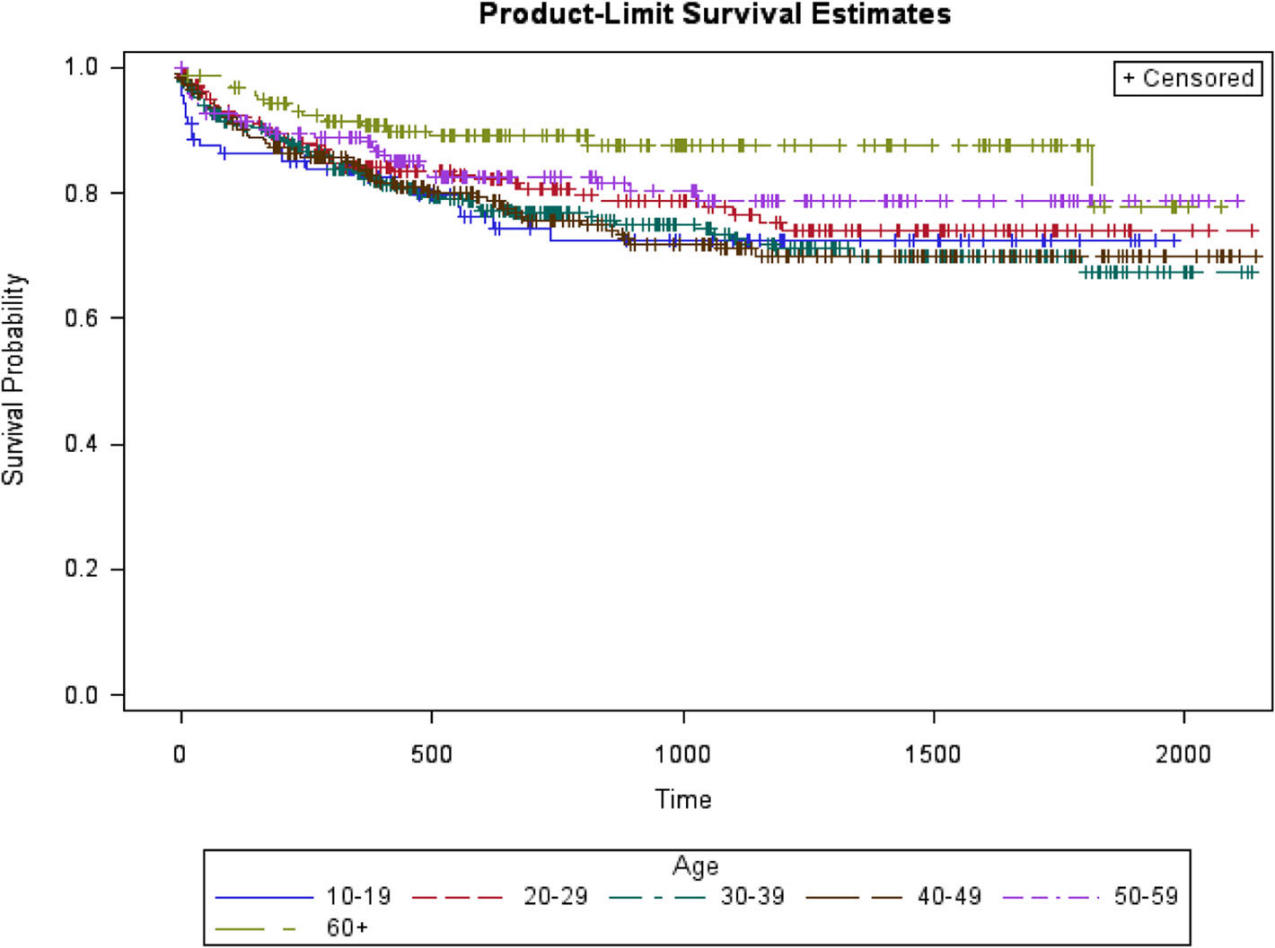
Survival estimates (re-attempts) considering age

**Fig. 5:**
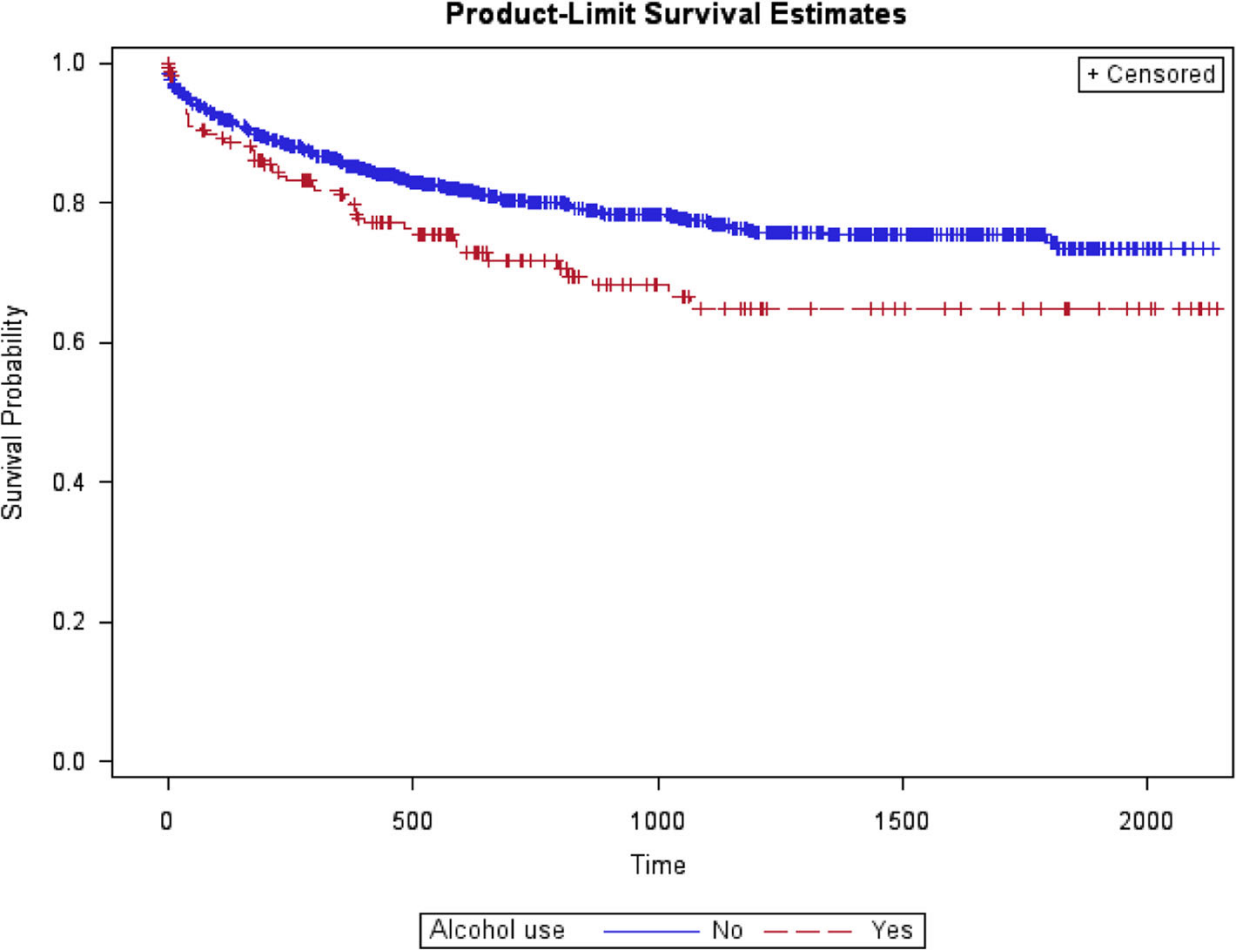
Survival estimates (re-attempts) considering alcohol use

**Fig. 6:**
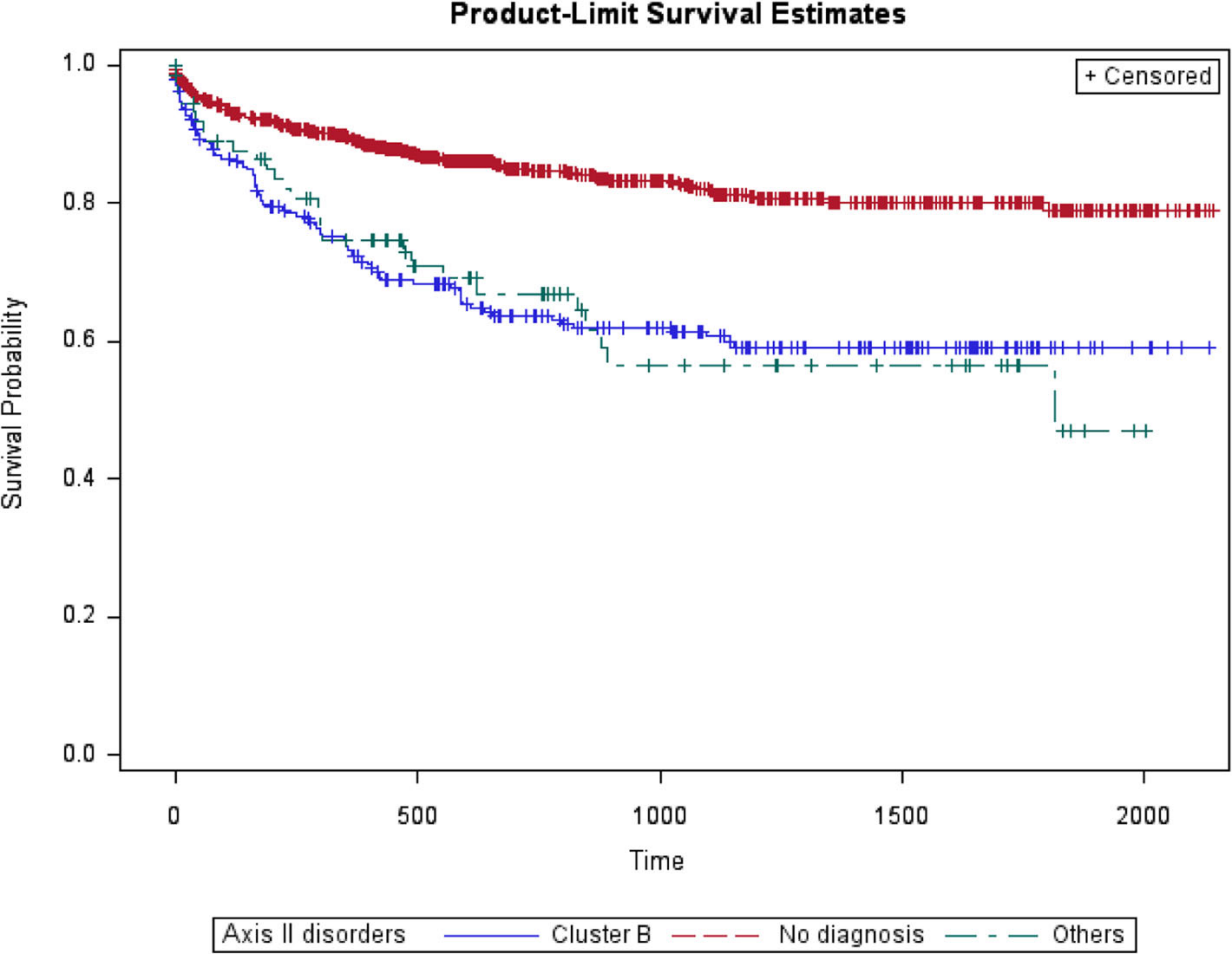
Survival estimates (re-attempts) considering personality disorders

As for the risk of re-attempts after the index suicide attempt, bivariate survival analyses showed that age, alcohol use and personality disorders presented differences in time to re-attempt (Figs. 4, 5, and 6). Compared with suicide attempters who re-attempt during follow-up, attempters who didn’t re-attempt were older. Indeed, being older than 60 years old was a protective factor. No statistically significant differences were observed in the rest of socio-demographic variables. Compared with non re-attempters, suicide re-attempters were also more likely diagnosed with cluster B personality disorders (36.8% vs. 16.6%; *p* < 0.001), and alcohol use disorders (19.8 vs. 13.9; *p* = 0.02).

All risk factors in bivariate analyses –age, alcohol use, and personality disorders- were entered into a multivariate model. Table 4 displays that all three factors contributed independently to increasing the risk for re-attempting suicide.

**Table 4:** Multivariate analysis for suicide attempt repetition

		*p*-value	H.R. (95% CI)
Age		0.0454	
	≤20		2.41 (1.27–4.58)
	20–29		1.76 (1.00–3.10)
	30–39		2.06 (1.22–3.51)
	40–49		2.09 (1.22–3.57)
	50–59		1.46 (0.80–2.68)
	≥60		-
Alcohol use		0.0111	
	Yes		1.52 (1.10–2.09)
	No		-
Personality disorder		<0.0001	
	No PD diagnosis		-
	Cluster B PD		2.47 (1.88–3.23)
	Other PD		2.66 (1.76–4.04)

Hazard ratios for the global follow-up period (*n* = 1207)

### Risk factors for suicide

Fifteen (*n* = 15) or 1.2% (15/1241) of first time suicide attempters evaluated eventually died by suicide. Ten (66.7%) were women. The index suicide attempt was mostly (80%) a drug overdose, the medical lethality was mild in 73.3% of cases, and 73.3% of cases were discharged after the index suicide attempt. The methods of suicide were jumping (*n* = 9), hanging (*n* = 5), and suffocation (*n* = 1). Most were depressed (6/15) or had no Axis I diagnosis (4/15) at baseline. Compared with suicide attempters who did not die by suicide, suicide attempters who did were more likely alcohol dependent (33.3% vs. 17.2%; *p* = 0.047), and older (50.9 ± 11.9 vs. 40.7 ± 16.0; *p* = 0.004). Nearly 90% [86.7% (3/15)] of suicides were aged 40 to 59 when they died. No other socio-demographic or clinical factors (either method used or severity of the index suicide attempt, being hospitalized after the index suicide attempt, or the presence of axis I or II diagnosis) were related to the risk of suicide death.

Of relevance, 86.7% (13/15) of the suicide attempters who eventually died by suicide did not complete all telephone follow-ups. Of these, four patients had already died by suicide when telephonically contacted for the first time, three of them during the first week in the aftermath of their evaluation in the ED, thus suggesting that the telephone call could not have done anything to prevent their suicide; six patients were lost to follow-up, and three patients rejected the follow-up because they were already being followed at a mental health clinic. On the other hand, 51.6% of those who did not suicide were followed-up until month 12 (see Table 5).

**Table 5:** Rate of suicide in suicide attempters who made or not the telephonic follow-up

	Completed suicide				
	Yes (*n* = 15)		No *(n* = 1226)		*p-value*
	*n*	%	n	%	0.0032
Follow up at 9 or 12 months	2	0.3%	633	99.7%	
Not follow up at 9 or 12 months	13	2.1%	593	97.9%	
Total (*n* = 1241)	15	1.2%	1226	98.8%	

## Discussion

In keeping with the literature, we found that younger age, and presence of personality disorders and alcohol use disorder were risk factors for re-attempting suicide in our sample of suicide attempters [10, 24, 25]. Furthermore, alcohol use and older age were risk factors for suicide. As for the telephone management program, around 50% of suicide attempters and 90% of those who died by suicide, respectively, did not complete the telephone follow-up at month 12. Those who completed the telephone follow-up were less likely to die by suicide. However, it was discouraging that most suicides (*n* = 127, nearly 90% of all suicides) were not evaluated in the ED during the study period.

Overall, there were more index suicide attempts for women than men in our sample, which is consistent with literature [26], and might be explained by the higher risk of depression among women [27]. Twenty percent of suicide attempters re-attempted suicide during the follow-up period. This is also in keeping with previous literature [13, 28]. In one study, 25% of the initial cohort of suicide attempters (*n* = 150; 38% had previous suicide attempts) re-attempted suicide during the 10-years follow-up [29]. In a Danish register-based survival analysis of 2614 suicide attempters matched with 39,210 non-attempters, 31.33% of suicide attempters re-attempted suicide within the follow-up period –average follow-up period was nearly 4 years- [10]. The authors stressed that the probability of suicide attempters re-attempting suicide was stronger during the first 2 years after the index suicide attempt. In a 10-year follow-up study between 1993 and 2002, from the initial 3690 suicide attempters admitted to Christchurch Hospital, 28.1% were readmitted for a further non-fatal suicide attempt [26]. Again, risk of readmission and rates of mortality from suicide were higher in the first 2 years after the index suicide attempt, but occurred throughout the 10 years follow-up period. The findings from both studies perfectly match our results as 88% of suicide attempters in our sample re-attempted within the first-2 years of follow-up.

Regarding risk factors for re-attempting suicide, we found three risk factors: 1) younger age; 2) presence of personality disorders; and 3) presence of alcohol use disorder, which is also in keeping with the literature. In the Danish registry-based survival analysis mentioned above [10], both younger age and alcohol abuse were risk factors for re-attempting suicide. Our results also match those reported by Osvath and colleagues when comparing first-time suicide attempters (*n* = 549) with repeaters (*n* = 609): both alcohol abuse, and particularly, the presence of personality disorders were associated with an increased risk of re-attempting suicide [30]. In a study following a similar case-control design comparing 112 first-time attempters and 159 repeaters, alcohol misuse was again one of the strongest factors associated with repetition of suicide attempts [31]. In a 20-year follow-up of first-ever suicide attempters, alcohol intoxication at index suicide attempt predicted repetition of suicide attempt at 5 years [32]. In the 10-year follow-up carried out by Gibb and colleagues, the factors associated with repetition were female gender, younger age, and use of a low-lethality suicide method [26]. In a previous study of 446 suicide attempters, we also reported that younger female attempters with severe personality disorders were prone to repeat suicide attempts [11].

On the other hand, 1.2% (15/1241) of suicide attempters evaluated died by suicide within the follow-up period, which is in the lower range of reported studies. For instance, in a follow-up of 11,563 patients who presented to hospital after deliberate self-harming, 1.5% and 3% died by suicide after 5 and 15 years of follow-up, respectively [33]. Also, in the 10-year follow-up mentioned above, of the initial 3690 suicide attempters admitted to Christchurch Hospital, 4.6% died by suicide [26]. In another cohort of 150 suicide attempters followed-up during 10 years in Catalonia, 12% completed suicide, and the risk was highest during the first 2 years after the index suicide attempt [29].

We found that the risk factors for suicide among suicide attempters followed-up in our study were: 1) being older; and 2) the presence of an alcohol use disorder. These results are also in keeping with literature [34–37]. Indeed, one of the most consistent findings in Suicidology is that suicide rates are higher among adults aged 60 and older [38]. Accordingly, suicide prevention programs should specifically be designed for this population [38]. On the other hand, alcohol use disorders not only increased the risk for re-attempting suicide but for suicide. Our finding is also in keeping with previous literature [39–41]. For instance, in a sample of 1018 unselected deliberate self-poisoning patients followed-up 14 years, of the 22.7% who had suicided by the end of the study, 85 (38.5%) showed clear evidence of long-term alcohol misuse [41]. These authors stressed that more attention should be paid to alcohol use disorders in suicide attempters [41].

However, our data cannot be generalized, as the most relevant and discouraging aspect of our study was that most suicides (127 out of 142, 87.5%) took place in people who were not evaluated in the ED during the study period. In other words, the 15 suicides among our sample of suicide attempters are likely not representative of suicide completers in our catchment area. For instance, most individuals who died by suicide in our study were women with a history of previous suicide attempts. However, most suicides within our catchment area, but who were not evaluated in the ED during the study period, were men (70.1%). Literature is clear in this respect: most suicides are male in most countries. Our finding that most suicides in our catchment area were not included in our sample might be explained by the fact that 60% of suicides in our area died during the first attempt, and 92.3% of suicides occurred during the first or second attempt [42]. Furthermore, most individuals who suicide are not followed up in mental health services, but rather in primary health services [5], thus making it difficult to identify individuals at risk.

Finally, 90% of those who eventually died by suicide, were not followed-up at month 12. This finding, paired with the above mentioned data that most suicides are followed up in primary health services, strongly suggests that, for the prevention of suicide, it is critical to implement “multiple practice improvements over several years” [16].

### Strengths and limitations

The major strength of our study is the sample size of suicide attempters, which allowed us to extract some valuable information on the risk of re-attempting suicide and suicide in suicide attempters. One limitation is that we did not individually follow-up our population during the 5-year period of study, only during the first year of telephone follow-up intervention, and instead relied on the electronic medical record of all suicide attempts evaluated at the ED. This means that suicide attempts that did not require medical intervention may have been missed. Nonetheless, it is likely we detected the medically severe suicide attempts. Moreover, we cannot rule out the possibility that some suicide attempts were evaluated in an ED at a different hospital during the follow-up. However, this possibility is unlikely because all severe suicide attempts are systematically referred to our hospital. Furthermore, the follow-up period (5 years) and the small number of suicides within the initial sample of suicide attempters limited our capacity to extrapolate the results to other populations of suicide. Finally, the most important limitation is that we could not explore the effectiveness of our telephone program in preventing suicide. This is important because most telephone preventive programs have been devoted to preventing re-attempts [20, 43, 44]. The sparse literature available on preventing suicide is not definitive. For instance, in a study examining long-term effects of a telephone helpline service, 18,641 services users were compared with a general population group in Italy [45]. They reported a reduction in suicide deaths among service users, but there was a lack of benefit for elderly males. Furthermore, a recent meta-analysis on the potential use of letters, green cards, telephone calls and postcards to preventing suicide did not find a “significant reduction in the odds of suicide in intervention compared with control” [46]. Accordingly, the authors recommended “further assessment of possible benefits in well-designed trials in clinical populations” before these brief interventions could be recommended for widespread clinical implementation.

## Conclusions

Younger age and the presence of either a personality disorder or an alcohol use disorder are risk factors for re-attempt in suicide attempters. Alcohol use and older age were risk factors for suicide. Most suicides within the period of study were not included in our study. Thus, our study raises an important question: longitudinal, follow-up studies are methodologically sound studies that allow drawing etiological connections between risk factors and suicide. The problem is that, if previously published follow-up studies on suicide had the same problem that we faced in our study –that most suicides were not “detected”-, most literature published to date on follow-ups could be extrapolating data from populations affected by a selection bias, thus not reflecting the real predictive characteristics of suicide deaths. Furthermore, research is clear on this: most who die by suicide do not even seek mental health services, and attempters and completes are different, although partially overlapping, populations [37, 47]. Thus, until we are able to detect most individuals at risk, and bearing always in mind that the prediction of suicide is impossible, probably the most intelligent interventions to decrease the daunting suicide rate are reducing access to means and a population-based strategy [47] directed to the prevention of depression in the general population by using different measures at different levels of the health system as recommended by the EAAD [16, 17, 48].

## Abbreviations

SB: Suicide behaviour
SIB: Suicidal ideation and behaviour
EAAD: European Alliance Against Depression
ED: Emergency Department
